# A new parameter describing fertility in rabbits at the farm level: the kit index

**DOI:** 10.5194/aab-61-463-2018

**Published:** 2018-12-06

**Authors:** Steffen Hoy

**Affiliations:** Department of Animal Breeding and Genetics, Justus Liebig University, Senckenbergstraße 3, 35390 Giessen, Germany

## Abstract

The kit index (KI) combines the kindling rate (KR) and the average litter size (LS) per group of does, per breeding round, per year, or per farm and represents the number of total or live-born kits per 100 inseminated does. The KR is the percentage of kindlings per number of inseminated does. Mathematically, the KI is the product of the KR multiplied by the average LS. The KI was calculated on the basis of 12 730 inseminated does and 89 864 live-born kits from one rabbit farm during a 25-month period from 2015 to 2017. The average KR was 70.1±9.1 % with a minimum (per breeding round) of 35.4 % and a maximum of 90.8 %. The average LS of total kits born was 9.16±0.91, whilst the average litter size of live-born kits was 8.87±0.90. The KI of total kits born was calculated to be 649± 121 kits per 100 inseminated does (min of 332, max of 971), while the KI of live-born kits ranged between 326 and 944 kits per 100 inseminated does (mean of 626±122). The KI is a normally distributed parameter with respect to both the total kits born and the live-born kits per 100 inseminated does. All three parameters (KR, LS, and KI) were characterized by large variations from week to week with a tendency toward a reduction in the summer months. No difference was found between the two housing units, but large differences were found between the two genetic strains used on the given farm. Therefore, it can be concluded that the KI is able to characterize the complex fertility situation on the given rabbit farm. The KI can be used to demonstrate and to solve problems regarding artificial insemination, in addition to general issues with insemination management.

## 1 Introduction

Following the guidelines of the International Rabbit Reproduction Group (IRRG) (International Rabbit Reproduction Group, 2005) the fertility of rabbit does is usually defined by the kindling rate (KR). Nevertheless, fertility can be estimated from the pregnancy rate using abdominal palpation (from the 10th day of pregnancy). The pregnancy rate (also known as the conception rate or fertility rate) characterizes the relationship between pregnant and inseminated does and includes uncertainties regarding gestation when detected by palpation. Prolificacy is considered as the litter size (LS) at birth (total and live-born kits) and should be recorded within a maximal interval of 24 h postpartum (International Rabbit Reproduction Group, 2005). This means that the fertility of does at the farm level is mainly characterized using the KR and LS parameters of total born or live-born kits. Further parameters describing the reproductive potential at the rabbit herd level are as follows: the number of litters per doe and per year; the average number of kits raised per litter, per doe, and per year; and the average weaning weight (Theau-Clément et al., 2006, 2015; Kumar et al., 2013; Coutelet, 2015). Schüddemage (1999), Carluccio et al. (2004) and Coutelet (2015) found respective pregnancy rates between 64 % and more than 80 % and an average LS of between 8.1 and 10.7 total kits born in different breeds. In practice, the KR is lower than the conception rate and varies widely between 29 % and 90 % (Dimitrova et al., 2009; Castellini et al., 2010; Manal, 2010; Gerencsér et al., 2011; Coutelet, 2015; Theau-Clément et al., 2016). It has been previously shown that housing and feeding are factors which influence reproductive performance. The LS of total kits born in modern hybrid strains varies between 9 and 12 kits (Coutelet, 2015; Maertens and Buys, 2015; Maertens and De Bie, 2017). Large differences between breeding rounds and seasons of the year have also been reported (Schüddemage, 1999; Matics et al., 2016). Fluctuations between breeding rounds can occur in both the KR and the LS, and may be either random or influenced by several factors. Small changes in both the KR and the LS parameters often remain unnoticed by the farm manager, but may significantly affect the total number of kits born per breeding round. The new rabbit kit index (KI) parameter combines the KR and the average LS per group of does, per breeding round, per year, or per farm and represents the number of total or live-born kits per 100 inseminated does. The aim of this study was to record the KR and LS parameters on a commercial rabbit farm over a longer period of time, in order to calculate the KI and determine possible factors influencing this parameter.

## 2 Materials and methods

### 2.1 Animals and materials

The analysis was carried out on a commercial rabbit farm situated in Baden-Württemberg, Germany. A total number of 12 730 inseminated does and 89 864 live-born kits were included in the study over a 25-month period from 2015 to 2017. The does were mainly kept in standard single cages with perforated plastic slatted floors, although since 2016 some does have been housed using a new “Combi system” (Meneghin, I). The cages were equipped with commercial feeders and nipple drinkers. An elevated platform (0.54×0.3 m plastic slatted floor) at a height of 30 cm and a standard nest box with straw or wood shavings, respectively, was available. The units (two identical housing units referred to hereafter as unit 1 and unit 2) on the farm were air-conditioned (16–21 ${}^{\circ }$C). Feed (standard pellets for does) and water were offered ad libitum. The duration of the light period was 16 h (from 06:00 to 22:00 local time). The rabbit kits were weaned at an age of 35 days. The does were artificially inseminated using a standard hormonal treatment (0.2 mL GnRH at artificial insemination) in a 42-day rhythm.

### 2.2 Methods

The documentation from the rabbit farm contained the following information: year, month, group, season of insemination (1 represents December–February; 2 represents March–May; 3 represents June–August; and 4 represents September–November), the number of inseminated does per group, the pregnancy rate (the number of pregnant does was assessed on the basis of abdominal palpation on day 18 of pregnancy), the kindling rate (percentage of does kindling related to the number of inseminated does), and the number of total kits and live-born kits per litter (International Rabbit Reproduction Group, 2005).

The KI combines the KR and the average LS per group of does (e.g., per breeding round, per season, per farm). Mathematically, the KI is the product of the KR multiplied by the average LS. The KR defines the actual percentage of does with a given litter based on the number of inseminated does; therefore, the KR is more precise than the pregnancy rate. In the present analysis, the KR, not the pregnancy rate, was used due to the uncertainties in the determination of pregnant does. The litter size with respect to total born and live-born kits was counted and registered for each doe after each kindling.

### 2.3 Statistical analysis

Statistical data analysis was carried out using SPSS version 23.0 for Windows. The descriptive statistics including the mean, the standard deviation (SD), and the min and max were calculated for the target parameters: the KR, the LS, and the KI of total born and live-born kits. The KI was tested for normality. Because the KI parameter was normally distributed, the differences between the genotypes and units were tested using a parametric test (Student's t test). Next, a univariate analysis of variance was calculated using the following linear model:

$$Y=\mu +{\text{genotype}}_{\text{a}}+{\text{unit}}_{\text{b}}+({\text{genotype}}_{\text{a}}x{\text{unit}}_{\text{b}}{)}_{\text{c}}+e_{\text{abc}},$$

with genotype (strain 2 and 4), unit (1 and 2), and the interaction between genotype and unit as fixed factors. The level of significance was set at P<0.05. It should be noted that no ethical consent was required. Data regarding the LS and KR were available from the commercial rabbit farm under farm conditions.

## 3 Results

Summarizing all data (n=12 730 inseminated does) the average pregnancy rate was 72.2±9.8 %; the minimum on the basis of breeding round was 36.6 % and the maximum was 90.8 %. The mean KR was calculated to be 70.1±9.1 % with a minimum (per breeding round) of 35.4 % and a maximum of 90.8 %. The average LS of total kits born was found to be 9.16±0.91, whilst the average LS of live-born kits was 8.87±0.90. The minimum LS of total kits born in one breeding round was 5.8 per litter, and the maximum was 10.9 per litter (on the basis of breeding rounds). The corresponding values for the LSs of live-born kits were 5.4 (minimum) and 10.7 (maximum) per litter.

The KI of total kits born was calculated as 649±121 kits per 100 inseminated does (min = 332, max = 971), whilst the KI of live-born kits ranged between 326 and 944 kits per 100 inseminated does (mean = 626±122) based on breeding rounds.

Significant differences were found between the two genetic strains with respect to all reproductive parameters. Does from strain 4 had a higher KR, a higher LS (total and live-born kits), and therefore also a higher KI than females from strain 2 (Table 1). Furthermore, the KR was 9 % higher, the LS was 1.1 (total kits) and 1.0 (live-born kits) greater, and the KI was therefore 156 higher, with respect to both total and live-born kits in does from genetic strain 4.

**Table 1:** Influence of genotype (strain) on reproductive parameters.

		N (breeding		
Parameter	Strain	rounds)	Mean	SD
Kindling rate (%)	2	33	65.7	9.5
	4	31	74.7	5.9
LS of total kits born	2	34	8.6	0.8
	4	33	9.7	0.6
LS of live-born kits	2	35	8.4	0.8
	4	33	9.4	0.6
KI of total kits born	2	32	572	102
	4	31	728	85
KI of live-born kits	2	33	551	102
	4	31	707	84

All differences in means are significant at the P<0.05 level. Differences in the calculation of KI are due to slightly different sample sizes and rounding.

The housing unit where does with kits were kept had no effect on the reproductive parameters. With respect to the tendencies of parameters, the KR was 2 % higher in unit 1. The mean LS of the total and live-born kits was nearly the same in both units. Therefore, the higher KR resulted in a KI that was 36 higher concerning the total number of kits born and 27 higher regarding the number of live-born kits per 100 inseminated does (Table 2).

**Table 2:** Reproductive parameters with respect to housing unit for does with kits.

		N (breeding		
Parameter	Unit	rounds)	Mean	SD
Kindling rate (%)	1	33	71.0	7.6
	2	31	69.0	10.5
LS of total kits born	1	35	9.2	1.1
	2	32	9.1	0.7
LS of live-born kits	1	36	8.9	1.1
	2	32	8.9	0.6
KI of total kits born	1	32	667	128
	2	31	631	115
KI of live-born kits	1	33	639	132
	2	31	612	110

All differences are significant at the P<0.05 level. Differences in the calculation of KI are due to slightly different sample sizes and rounding.

**Table 3:** Influence of genotype (strain) on reproductive parameters – separated for both units.

			N (breeding		
Unit	Parameter	Strain	rounds)	Mean	SD
1	Kindling rate (%)	2	16	66.1	6.2
		4	17	75.6	5.8
	LS of live-born kits	2	18	8.2	1.1
		4	18	9.5	0.7
	KI of live-born kits	2	16	546	101
		4	17	727	92
2	Kindling rate (%)	2	17	65.3	12.0
		4	14	73.5	6.1
	LS of live-born kits	2	17	8.5	0.5
		4	15	9.3	0.5
	KI of live-born kits	2	17	555	107
		4	14	682	67

All differences between strains in both units are significant at the p<0.05 level. Differences in the calculation of KI are due to slightly different sample sizes and rounding.

The does from genetic strain 4 displayed better performance with respect to the KR, the LS of live-born kits, and the KI in both units 1 and 2 compared with their pen mates from strain 2 (Table 3).

**Figure 1:**
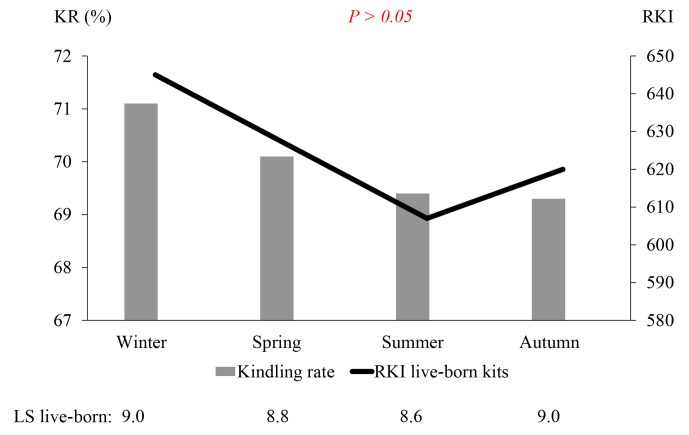
Kindling rate, mean litter size of live-born kits, and the kit index (KI) with respect to the season of the year.

With regards to tendency, the season of the year had a nonsignificant effect on the KR, the LS (total and live-born kits), and the KI (Fig. 1). The highest KR (71.1 %), and therefore the highest KI (645 live-born kits per 100 inseminated does), was found in winter (kindling in December–February). The lowest KR and KI were found in summer (June to August) and autumn (September to November). In these seasons the KR was 69.4 (summer) and 69.3 (autumn), whilst the KI was 607 (summer) and 620 (autumn). The lowest LS of live-born (and also total) kits occurred in summer (June–August) and was 8.6±0.8, whilst the highest LS was found in both autumn and winter (9.0±0.8).

**Figure 2:**
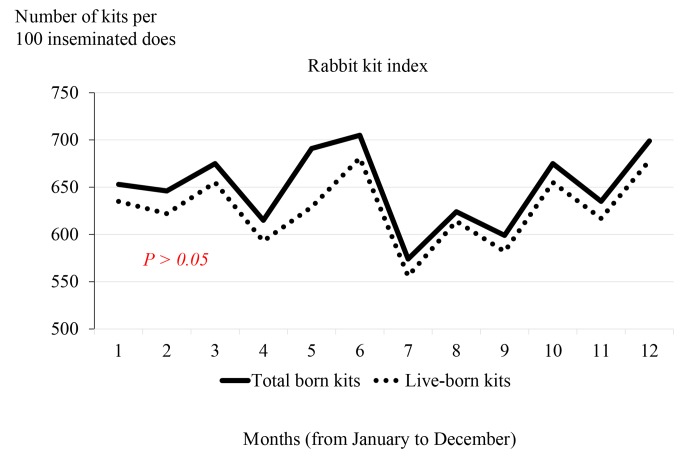
The rabbit kit index of total born kits and live-born kits with respect to month of the year.

On the basis of month, the highest KI (total and live-born kits per 100 inseminated does) was found in June (705 and 681 for total and live-born kits, respectively) and the lowest was seen in July (574 and 556, respectively) (Fig. 2). There is a large fluctuation in the KI between months. It seems that the fluctuations from month to month in late spring and summer are larger than in the other months. With respect to the tendency of KI, this parameter decreases from July to September, before increasing again in autumn and winter.

## 4 Discussion

The rabbit kit index (KI) introduces a new parameter that combines the kindling rate (KR) and the litter size (LS). Small variations in both parameters multiply to considerable differences, e.g., between genotypes, breeding rounds, seasons, or probably also farms. The following example demonstrates the effect of KI:

- On a (fictitious) rabbit farm a KR of 70 % and an average LS of 9.2 total kits born are achieved resulting in a KI of 644 total kits born per 100 inseminated does.
- The 2 % improvement of the kindling rate (with the same LS = 9.2) leads to a KI of 662.
- The 0.2 increase in the mean number of total kits born (with the same KR of 70 %) results in a higher KI of 658.
- Improving the kindling rate by 2 % as well as increasing the litter size by 0.2 leads to a 33 kit per 100 inseminated does (KI = 677) increase in KI.

In our analysis, the average pregnancy rate was 72.2 %. This corresponds well with the average value (approximately 70 %) found in the literature (Carluccio et al., 2004; Coutelet, 2015). The KR (on average 70.1 %) was consequently 2.1 % lower. In the given analysis, the KR was also lower than that reported by Castellini et al. (2010), Manal (2010), Gerencsér et al. (2011), Coutelet (2015), and Theau-Clément et al. (2016). Furthermore, the LS was relatively low compared with results from the literature concerning the breeding goals of modern hybrid rabbit strains (Coutelet, 2015; Maertens and Buys, 2015; Maertens and De Bie, 2017).

This study represents the first time that the new KI parameter has been calculated. The differences between the breeding rounds concerning KI were found to be extremely high ranging from 332 to 971 (total kits born per 100 inseminated does) and from 326 to 944 (live-born kits per 100 inseminated does). This means that in 1 week 332 kits from 100 inseminated does were born (number of total kits born) and in another week 997 kits from 100 inseminated does were born. These differences seem more powerful than the differences in the KR and LS alone. It was not possible to clarify probable causes for these major differences, as this study was a retrospective evaluation under the real-world conditions of a commercial rabbit farm. In general, the housing conditions were not changed throughout the experimental period. Furthermore, the people who inseminated the does were the same during this period of time. It is common knowledge that rabbit fertility is influenced by the season of the year, the microclimate, day (light) length, the animals' health situation, several nutritional factors, and general farm management (Theau-Clément et al., 2006, 2015; Huneau-Salaün et al., 2015). One of the factors that has a large impact on the KI tendency is the season or the outdoor and indoor temperature. The lowest KR and LS, and thus the lowest KI, was found in summer (Fig. 1) with extreme data being noted in July: the KI was particularly low in July (Fig. 2). High temperature values during the summer are a major factor constraining rabbit production, as heat stress affects production (Fouad, 2005; Yassein et al., 2008). Kumar et al. (2013) found that hot summer temperatures had a significant impact on the LS, whereas the hot Algerian summer season did not seem to affect fertility parameters (Zerrouki et al., 2005).

The genetic strain of the females, the doe replacement strategy, and the nursing practices appeared to significantly influence reproductive performance (Huneau-Salaün et al., 2015). Indeed, highly significant differences were found in our analyses between genetic strains 2 and 4 which were used on the farm. Doe replacement and nursing strategy were not changed during the data collection period. Following Huneau-Salaün et al. (2015), maternity management seems to be the most important point in rabbit unit management with respect to improving the productivity of rabbit farms. In our study, according to the farm manager, problems with feed quality and issues with sperm doses from a commercial extender (problems with extender) during production may have affected the fertility parameters in summer 2017. Therefore, problems with management and the environmental load can be evaluated using the KI parameter.

The KI for the total kits born directly represents the result of artificial insemination. Even stillborn kits resulted from fertilized ova. The KI could also be used in natural mating; however, for a reasonable application at least 40 to 50 does would need to be mated at the same time. The KI for the live-born kits includes factors that influence kindling and early mortality. Both parameters can be used to assess reproductive management.

## 5 Conclusions

The rabbit kit index (KI) is a new and innovative parameter that combines the effects of the kindling rate (KR) and the litter size (LS, total or live-born kits) and indicates the number of total or live-born rabbit kits per 100 inseminated does. Mathematically, the KI is the product of the KR multiplied by the average LS. It may be able to contribute to the improvement of reproductive performance on rabbit farms.

## Supplement

10.5194/aab-61-463-2018-supplement The supplement related to this article is available online at: https://doi.org/10.5194/aab-61-463-2018-supplement.

## Data Availability

The data used in this study are available in the Supplement (file data kit index).
