# Supplementary material for: A new parameter describing fertility in rabbits at the farm level: the kit index
Source: Arch Anim Breed. 2018 Dec 6;61(4):463–7. doi: 10.5194/aab-61-463-2018 (PMC7065404; doi:10.5194/aab-61-463-2018)
Supplement: The supplement related to this article is available online at: https://doi.org/10.5194/aab-61-463-2018-supplement. [file aab-61-463-supplement.zip › aab-61-463-2018-supplement-title-page.pdf]

Supplement of Arch. Anim. Breed., 61, 463–467, 2018
https://doi.org/10.5194/aab-61-463-2018-supplement
© Author(s) 2018. This work is distributed under
the Creative Commons Attribution 4.0 License.

Open Access
Archives Animal Breeding

Supplement of

A new parameter describing fertility in rabbits at the farm level: the kit index

Steffen Hoy

Correspondence to: Steffen Hoy (steffen.hoy@agrar.uni-giessen.de)

- aab-61-463-2018-supplement-title-page.pdf
- aab-2018-43-supplement.xlsx

The copyright of individual parts of the supplement might differ from the CC BY 4.0 License.
